# Supplementary material for: Reduction of Influenza A Virus Prevalence in Pigs at Weaning After Using Custom-Made Influenza Vaccines in the Breeding Herds of an Integrated Swine Farm System
Source: Viruses. 2025 Feb 10;17(2):240. doi: 10.3390/v17020240 (PMC11860655; doi:10.3390/v17020240)

Supplemental Figure S1: H3 phylogenetic tree. Phylogenetic tree constructed with obtained H3 sequences from farms. In black fonts: reference Genomes; red font: circulating strains; purple font: vax strains

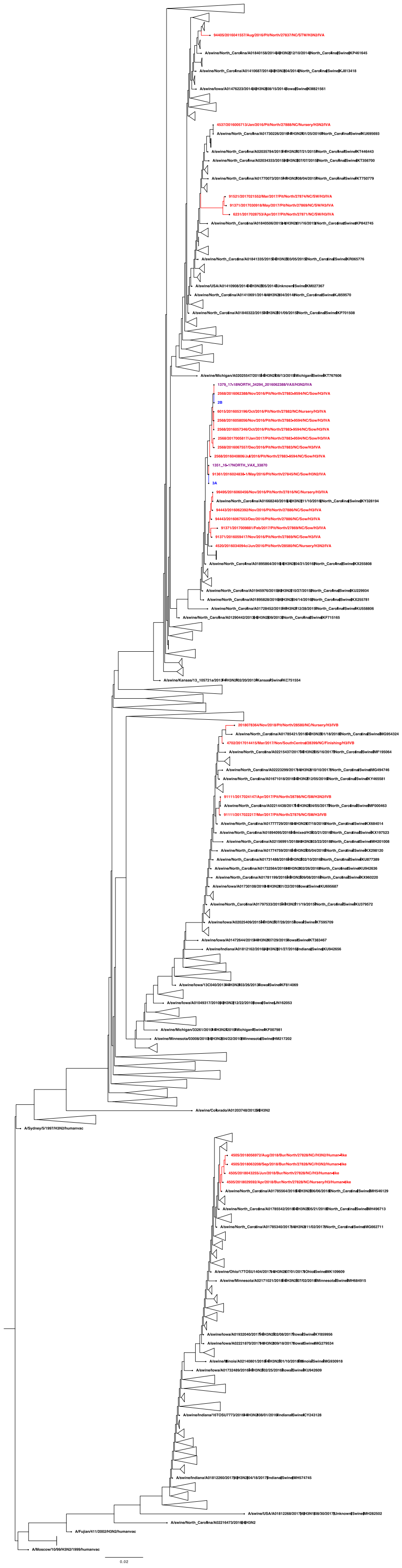

Supplemental Figure S2: H1 phylogenetic tree. Phylogenetic tree constructed with obtained H1 sequences from farms. In black fonts: reference Genomes; red font: circulating strains; purple font: vax strains

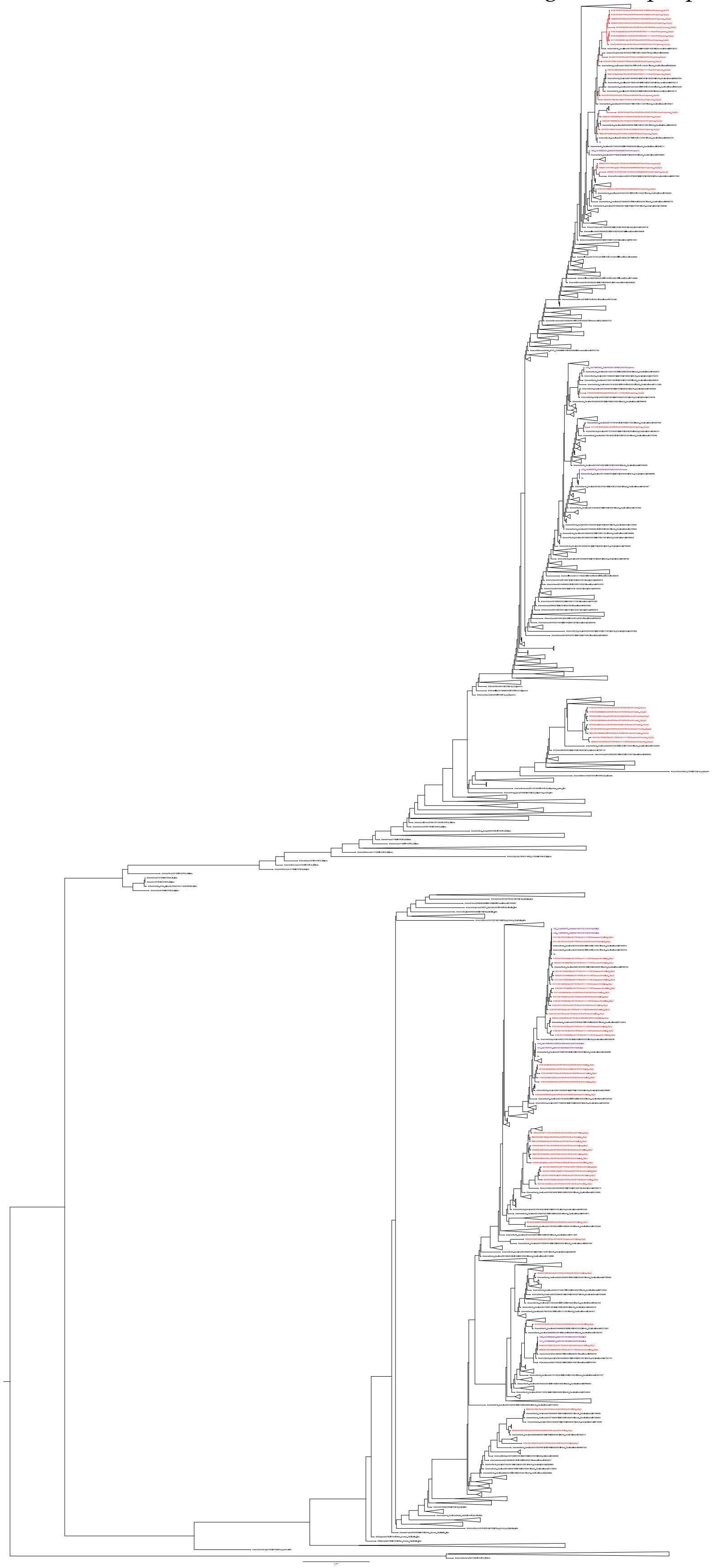

Supplement: Supplementary file 1 [file viruses-17-00240-s001.zip › viruses-3429868-supplementary.pdf]
